# Sodium and Potassium Intake Assessed by Spot and 24-h Urine in the Population-Based Tromsø Study 2015–2016

**DOI:** 10.3390/nu11071619

**Published:** 2019-07-16

**Authors:** Haakon E. Meyer, Lars Johansson, Anne Elise Eggen, Heidi Johansen, Kristin Holvik

**Affiliations:** 1 Department of Chronic Diseases and Ageing, Norwegian Institute of Public Health, 0213 Oslo, Norway; 2 Department of Community Medicine and Global Health, Institute of Health and Society, University of Oslo, 0318 Oslo, Norway; 3 Department of Community Medicine, UiT The Arctic University of Norway, 9037 Tromsø, Norway

**Keywords:** salt, sodium, potassium, 24-h urine, spot urine, population-based study

## Abstract

Reduction of salt intake is a public health priority and necessitates the surveillance of salt intake in the population. The validity of salt intake assessed by dietary surveys is generally low. We, therefore, aimed to estimate salt intake by 24-h urine collection and to assess the usefulness of spot urine collection for surveillance purposes. In the population-based Tromsø Study 2015–2016, 493 men and women aged 40–69 years collected 24-h urine, of whom 475 also collected spot urine. Sodium and potassium excretions were calculated by multiplying respective urinary concentrations by the total volume of urine. Based on the sodium concentration in spot urine, we also estimated 24-h sodium excretion by three different equations. Mean sodium excretion was 4.09 ± 1.60 and 2.98 ± 1.09 g/24-h in men and women, respectively, corresponding to a calculated salt intake of 10.4 and 7.6 g. The sodium to potassium molar (Na/K) ratio was approximately 1.8 in both genders. Of the three equation utilizing spot urine, estimated mean 24-h sodium excretion was closest for the INTERSALT formulae (4.29 and 2.96 g/24-h in men and women, respectively). In this population-based study, the estimated salt intake was higher than the recommended intake. However, urine potassium excretion was rather high resulting in a favorable Na/K ratio. Mean sodium excretion calculated from spot urine by the INTERSALT equation predicted the mean sodium excretion in 24-h urine reasonably well.

## 1. Introduction

High dietary sodium and low dietary potassium intakes are associated with hypertension and increased risks of cardiovascular diseases (CVD) [1,2,3,4]. As a contributor to the global burden of disease [5,6,7], the World Health Organization (WHO) and several countries have implemented policies to reduce the sodium intake of their populations [8,9], including Norway [10]. WHO recommends an intake of <2 g of sodium per day (corresponding to 5 g NaCl (salt)) [8]. In the Nordic recommendations from 2014 [11] and the US National Academies of Sciences, Engineering, and Medicine recommendations from 2019 [12], a limitation of sodium intake to approximately 2.3 and 2.4 g per day (corresponding to approximately 6 g salt) is recommended. Surveillance of intake of sodium, potassium and several other dietary components are important for targeting and follow-up of public health nutrition policies [10].

Estimating sodium intake by dietary survey methods is difficult and the validity is generally low [13,14,15]. The preferred method for estimating daily sodium intake is 24-h urine collection [16,17]. Similarly, the amount of potassium excreted in 24-h urine is correlated with dietary potassium intake [17,18]. However, collecting 24-h urine specimens is often not feasible, while spot urine is easier to obtain. Prediction models for estimating 24-h urine sodium and potassium excretion from casual spot urine samples have been developed and evaluated [19,20,21]. A systematic review concluded that estimating mean population sodium intake from spot urine samples can provide good indications of mean sodium intake at the population level, although 24-h urine collection from a subset is recommended for the purpose of tracking changes in intake over time [22].

In the closely monitored Tromsø population in Northern Norway, a substantial decrease in blood pressure has been reported over recent decades [23], but salt intake has not been studied. In 2015–2016, 24-h and spot urine were collected in a subsample of the cohort, and the aim of the present study was to (a) assess sodium and potassium excretion and sodium to potassium molar ratio (Na/K ratio) in 24-h urine and further estimate the intake of salt in the adult Tromsø population and (b) compare urinary sodium, potassium and Na/K ratio in 24-h urine and casual spot urine, and evaluate if spot urine collection may be useful for surveillance purposes.

## 2. Materials and Methods

The Tromsø Study is a population-based, prospective study conducted in Tromsø, Northern Norway by the University of Tromsø. It was initiated in 1974 as a combined population health survey and a research study of cardiovascular diseases, and it has gradually expanded to include several chronic diseases. Seven surveys have been carried out 6–7 years apart, referred to as Tromsø 1–7. All surveys include questionnaire data, sampling of biological specimens, and clinical measurements.

The seventh wave of the Tromsø Study (Tromsø 7) was carried out during 2015–2016. All citizens 40 years and older in Tromsø were invited, and 21,083 participated (participation rate 65%) (www.Tromsostudy.com). After excluding persons with known heart or liver failure, cerebral stroke, liver disease and participants who had started treatment with diuretics during the preceding two weeks and participants with conditions making it difficult to collect urine (e.g., wheelchair, poor general condition), a random sub-sample of 608 attendees aged 40–69 years were invited for the current substudy. Of these, 496 (82%) agreed to participate and collected urine in the period August 2015–April 2016. Approximately half of the 24-h urine collections were made on weekdays and the remainder on weekends. Three persons with undetermined urine volume were excluded from the analyses. Of those included, 475 also provided a spot urine sample. Whereas men and women collecting 24-h urine were 2.9 years (*p* < 0.001) and 1.6 years (*p* = 0.003) older than men and women 40–69 years not collecting urine, there were no statistical significant difference in BMI or education between those collecting and not collecting urine. The participants were given oral and written instruction for collection and handling of all urine during a 24-h period. The participants were instructed to empty their bladders the first morning and discard this urine, and subsequently collect all urine during the next 24 h, including the morning void the day after. Participants were asked to record the start and end time as well as any irregularities regarding their collection of urine. Containers with urine were kept cool during urine collection.

The participants were also instructed to collect a sample of spot urine at any time during the 24-h period. The volume of this sample also counted in the calculation of 24-h urine volume.

After returning the 24-h urine specimens, the containers were well stirred and volume was read and recorded. Urine samples of 2 ml were extracted and stored at −20 °C until analysis.

All analyses of the samples were done at the Department of Laboratory Medicine, University Hospital of Northern Norway, UNN, Tromsø, Norway at the same time. Urinary sodium and potassium were assessed using Roche Hitachi—an indirect ion-selective electrode to determine ion concentration. Urinary creatinine was assessed using Cobas 8000/Roche by an enzymatic colorimetric method. The Department of Laboratory Medicine was accredited by Norwegian Accreditation according to the standard NS-EN ISO 15189 TEST 209.

Urinary concentrations of sodium, potassium, and creatinine were determined in mmol/L, and we used the conversion factor to grams of 0.023 for sodium and 0.039 for potassium. Daily excretions of sodium and potassium were calculated by multiplying respective urinary concentrations by the total volume of urine. Estimated daily salt intake in grams was subsequently calculated by multiplying daily sodium excretion by 2.54 [22].

Na/K ratio was calculated from 24-h urine and spot urine samples by dividing the concentration of sodium by the concentration of potassium, both in mmol/l. We estimated 24-h sodium excretion from spot urine by equations developed by the INTERSALT group [19], Tanaka and coworkers [21] and an equation developed in a Danish study population by Toft and coworkers [20]. These equations predict 24-h sodium excretion using age, anthropometric measures, and spot urine concentration of sodium and creatinine (and potassium for the INTERSALT equation). For the INTERSALT equation, we used the version applicable to the Northern European region [19]. Details are given in supplementary Table S1. The frequently used Kawasaki formula is not included in our manuscript as this formula was developed for urine collected at the second-morning void.

Differences according to gender and educational were tested by independent samples T-test and ANOVA. In additional analyses, we tested the gender differences by the non-parametric Mann-Whitney U Test. Adjustment for age and body weight was done by ANCOVA and linear regression. Paired samples T-test was used to compare the results from the 24-h urine and spot urine samples. For the comparisons of spot urine and 24-h urine, we also calculated Pearson correlation coefficients, and Bland-Altman plots were constructed to evaluate whether the difference between sodium excretion from spot urine and 24-h urine varied across individual mean sodium excretion from spot urine and 24-h urine.

Additional analysis was performed excluding those who reported ≤ 17 h or ≥ 31 h of urine collection, those who did not report the duration of urine collection, and those who reported that their urine collection was incomplete (n = 101), leaving n = 392 for this sensitivity analysis.

### Ethical Considerations

The Norwegian Data Protection Authority and the Regional Committee of Medical and Health Research Ethics, North Norway approved Tromsø 7. The study complies with the Declaration of Helsinki, International Ethical Guidelines for Biomedical Research Involving Human Subjects and the International Guidelines for Ethical Review of Epidemiological Studies. Participation was voluntary and each subject gave written informed consent prior to participation. This substudy was approved by the Regional Committee of Medical and Health Research Ethics (REK 2016/1795).

## 3. Results

Mean 24-h urine volume was approximately 1.7 L in both genders (Table 1). Men had higher sodium and potassium excretion than women. However, the Na/K ratio was in the magnitude of 1.8 in both genders. Based on 24-h sodium excretion, mean sodium intake was calculated to be 4.09 g/day in men and 2.98 g/day in women, corresponding to 10.39 g of salt in men and 7.55 g in women. Median calculated salt intake was somewhat lower, i.e., 9.73 g in men and 7.13 g in women. The distribution of calculated salt intakes is shown in Supplementary Figure S1. A sodium excretion corresponding to a salt intake under 6 g was found in 13% of the men and 29% of the women. After adjusting for body weight, the mean gender difference in calculated salt intake was reduced from 2.84 g to 1.48 g.

Sodium and potassium excretion did not differ significantly across age groups in men, whereas women aged 55–69 years had a lower excretion of sodium compared to younger women (Table 2). Older women had also a significantly lower Na/K ratio compared to younger women.

In men, higher education was associated with a higher potassium excretion and a lower Na/K ratio, whereas sodium excretion did not differ significantly across educational groups (Table 3). In women no statistically significant educational differences were found in sodium or potassium excretion, although a somewhat similar pattern as for men was suggested. The educational differences became moderately stronger by adjustment for age (data not shown), with a statistically significant decrease in Na/K ratio with higher education in women (*p* = 0.03).

### 3.1. Spot Urine versus 24-h Urine Samples

Among the 475 participants who provided a spot urine sample, the concentration of both sodium and potassium was significantly higher in spot urine as compared to 24-h urine in women, but not in men (Table 4). On the other hand, the Na/K ratio was similar in spot and 24-h urine in both genders. Stratified analyses in women indicated that sodium and potassium was higher in spot urine compared to 24-h urine both in those collecting urine before and after noon.

The Pearson’s correlation coefficient for the Na/K ratio from 24-h urine and spot urine was r = 0.47 in men and r = 0.65 in women (Supplementary Figure S2). When excluding outliers with Na/K ratio >4, it changed to r = 0.56 in men and r = 0.61 in women.

Compared to sodium excretion in 24-h urine samples, sodium excretion during 24 h estimated by the INTERSALT formula was closest both in men and women and performed better than daily sodium estimated by the formulae developed by Toft and coworkers [20] and Tanaka and coworkers [21] (Table 5). Mean daily sodium excretion estimated by the INTERSALT formula was 4% higher in men and 1% lower in women than that measured by 24-h urine. Converted to salt intake this difference corresponds to 0.4 g/day for men (10.9 g vs. 10.5 g, *p* = 0.065) and −0.1 g/day for women (7.5 g vs. 7.6 g, *p* = 0.59). The corresponding correlation coefficients were r = 0.59 in men and r = 0.57 in women. Bland–Altman plots showed that daily sodium excretion estimated from spot urine was overestimated at low sodium excretion and underestimated at high sodium excretion (Supplementary Figure S3).

### 3.2. Sensitivity Analyses

Excluding persons who stated that they had collected urine for less than 17 h or more than 31 h, persons who did not state how many hours they had collected urine and persons stating that they had not collected all urine, in total 101 persons, hardly affected the calculated mean sodium excretion (178.1 after exclusion versus 177.9 mmol/24-h before exclusion in men and 128.9 versus 129.4 mmol/24-h in women). The same was the case for 24-h sodium excretion estimated from the INTERSALT equation (184.4 versus 186.5 mmol/24-h in men and 127.1 versus 128.2 mmol/24-h in women).

## 4. Discussion

In the adult Tromsø population, we found that salt intake estimated by 24-h urine excretion, especially among men, was higher than the officially recommended intake. However, urine potassium excretion was rather high, resulting in a favorable Na/K ratio in both men and women. Sodium concentrations in spot urine predicted the mean daily sodium excretion reasonably well in the Tromsø population when using the INTERSALT equation.

### 4.1. Sodium Excretion

The mean 24-h sodium excretion of 3.53 g/day in the Tromsø population (men and women combined) was in the lower range of the distribution of 24-h sodium excretion reported in a systematic review from 2013 of 51 studies from Western Europe [24]. They reported that mean sodium intakes across studies ranged from 3.28 to 4.43 g/day, in both genders combined. The mean 24-h sodium excretion of 4.09 g/day in men and 2.98 g/day in women in our study is comparable to that recently reported for US adults in the NHANES 2014 study (4.2 g/day in men and 3.0 g/day in women) [25], and lower than reported for men and women in Italy (4.4 and 3.4 g/day) [26] and Greece (4.5 and 3.6 g/day) [27]. On the other hand, 24-h sodium excretion in our population was higher than that reported for men and women in Finland in 2002 after two decades with a salt reduction program (3.7 and 2.8 g/day) [28] and New Zealand (3.9 and 2.9 g/day) [29]. Especially in men, intake in Tromsø was higher compared to Somali immigrants living in Oslo, who had a 24-h sodium excretion of 3.5 g in men and 2.9 g in women [30].

The sodium excretion in Tromsø 2015–2016 was lower compared to historic Finnish data from 1982 (around 5.0 g/day in men and 3.4 g/day in women) [28] and among men in a population-based survey in western Norway around 1980 (4.4 g/day) [31].

### 4.2. Potassium Excretion

Interestingly, mean 24-h potassium excretion of 3.87 g/day in men and 2.97 g/day in women was substantially higher than that reported in several other populations such as men and women in the US (2.40 and 1.92 g/day) [25], Italy (2.46 and 2.15 g/day) [26], Greece (2.76 and 2.36 g/day) [27], New Zealand (3.0 and 2.4 g/day) [29] and Finland (3.17 and 2.68 g/day) [28]. It was also higher than potassium excretion in Somali men and women in Oslo (2.61 and 2.14 g/day) [30] and in men in western Norway in 1980 (3.3 g/day) [31].

### 4.3. Sodium-to-Potassium Ratio

The mean Na/K ratio of 1.86 in men and 1.79 in women measured in our study was substantially lower than the ratio reported for men and women in the NHANES 2014 study (3.17 and 2.87) [25], and lower than that reported for men and women in Finland (2.08 and 1.92) [28], New Zealand (2.3 and 2.1) [29], Somalis in Oslo (2.5 and 2.4) [30], Greece (2.87 and 2.77) [27] and Italy (3.1 and 2.8) [26].

Mean Na/K ratio was similar in 24-h urine and spot urine, indicating that the Na/K ratio from spot urine performed well at the population level in our study population. However, the correlation between the two was moderate, and as can be seen in Supplementary Figure S2, there were large variations in Na/K ratio in spot urine at a given level of Na/K ratio in 24-h urine. One possible explanation for this could be diurnal variations in excretions [32].

### 4.4. Sodium, Salt, and Potassium: Comparison with Recommended Intake

In Tromsø, the calculated mean salt intake of 10.4 g/day in men and 7.6 g/day in women was substantially above the officially recommended level of 6 g/day. It could be added that in the Global Burden of Disease Study, 3 g of sodium (corresponding to 7.6 g of salt) was used as the optimal level of intake in their estimations of health effects of dietary risks [7].

The daily salt intake calculated by urine excretion is a conservative estimate of salt consumption as there are, in addition, non-urinary losses (e.g., sweat) and approximately 90% of dietary intake is excreted in the urine [12,24].

It is often indicated that around three-quarters of the dietary intake of potassium is excreted in the urine, but this may vary considerably and may be influenced by other dietary intakes [12]. Using 24-h potassium excretion as a conservative estimate of intake, the magnitude of mean potassium excretion of 3.9 g/day in men and 3.0 g/day in women corresponds closely to the Nordic recommended intake level for men (3.5 g/day) and women (3.1 g/day) [11], and the 3.5 g/day recommended by the WHO [33]. Furthermore, it is substantially higher than the adequate intake (AI) level of potassium of 3.4 g/day for men and 2.6 g/day for women recently set by the US National Academies of Sciences, Engineering, and Medicine [12]. Our data suggest that the recommended intake level of potassium is met among Tromsø inhabitants. Our results are in line with findings from nationwide dietary surveys among adults, suggesting that the habitual intake of potassium in men and women is higher in Norway (4.2 and 3.4 g/day) [34]) compared to several other countries such as the US (3.0 and 2.3 g/day) [35], the UK (3.1 and 2.6 g/day) [36], Sweden (3.4 and 2.9 g/day) [37], and the Netherlands (3.9. and 3.0 g/day) [38]. 

A high intake of potassium has been linked to lower blood pressure and reduced risk of CVD [33], although it is claimed that the beneficial effects of potassium could in part be ascribed to other beneficial nutrients in the same foods supplying potassium, e.g., fruits and vegetables [12].

Specifically, the combination of low sodium intake and high potassium intake expressed by the Na/K ratio has been studied as a predictor for reduced CVD risk [39] and may predict CVD risk better than sodium and potassium intakes separately. According to WHO, the optimal Na/K ratio would be approximately one if the recommended intakes are fulfilled [33]. If the Tromsø population in the future manages to reduce its salt intake to less than 6 g per day while maintaining their relatively high potassium intake, the Na/K ratio will move below one. Although the Na/K ratio in our study was well above one, it was more beneficial than reported from many other populations. On the other hand, in the recently updated US Dietary Reference Intakes for sodium and potassium it was concluded that the evidence is currently insufficient to establish recommendations regarding the Na/K ratio [12].

### 4.5. Variation by Age, Gender, and Education

A higher average sodium and potassium consumption and excretion are expected in men compared to women due to higher food and energy intake. In the Norwegian national dietary survey men on average had 36% higher energy intake and 24% higher potassium intake compared to women [34]. Correspondingly, in our study both sodium and potassium excretion were higher in men than women, while the Na/K ratio was similar.

In Tromsø, sodium and potassium excretion did not differ significantly between men aged 40–54 or 55–69 years. Interestingly, women aged 55–69 years had lower excretion of sodium and a lower Na/K ratio compared to younger women. The reason for this is not clear. It could be due to lower energy intake in older women, but they also had a higher potassium intake compared to younger.

There is a well-known socioeconomic gradient in health, and higher rates of hypertension and CVD have been reported in groups with lower education [40]. Correspondingly, we found that men with shorter education had a lower potassium excretion and a higher Na/K ratio compared to men with longer education.

### 4.6. Spot Urine and Estimated Sodium Intake

Several equations have been developed to estimate salt intake from spot urine samples. We used the frequently used INTERSALT equation [19], the TANAKA equation [21], and an equation developed in Denmark, a neighboring Nordic country [20]. Of these, the INTERSALT equations worked best in our population, and the mean difference between salt intake estimated from INTERSALT spot urine and 24-h urine of 0.4 g/day for men and −0.1 g/day for women is close to the difference of −0.4 g/day (both genders combined) presented in a meta-analysis [22]. It is important to keep in mind that these equations at their best give estimates of mean intakes and can lead to large misclassifications when estimating individual intakes [41]. As previously reported [12,22], and as found in our study, these equations tend to overestimate excretion at low intakes and underestimate excretion at high intakes. Due to this, estimating intake in a population by these equations can lead to over- or underestimation of salt intake. It has been recommended to obtain 24-h urine samples in a subset, especially when aiming to detect changes in mean salt intake in a population over time [22]. On the other hand, estimated mean sodium excretion from spot urine by the INTERSALT equation predicted the mean sodium excretion in 24-h urine reasonably well in our population as the mean intake was at a point with no under- or overestimation (Figure S3).

### 4.7. Strengths and Limitations

A strength of this study was the population-based sample with a reasonably large sample size and high participation rate. Data on sodium excretion from 24-h urine collection in population-based studies are relatively scarce, and as far as we are aware, there is only one previous Norwegian population-based study performed in men nearly 40 years ago [31]. Another strength is the concurrent collection of spot urine samples. Although the collection of 24-h urine is a strength, it is a limitation that it was only collected once in each participant. In order to classify individuals better, repeated 24-h collections should be done [12]. There is a substantial day-to-day variation in sodium and potassium excretion [32,42], and more than four collections have been recommended in order to assess individual intakes [42]. In addition, with only one urine collection, the standard deviation will increase (random error), inflating the proportion of the participant with values above or below given cut points [12].

## 5. Conclusions

We found that the salt intake estimated from 24-h urine excretion was higher than the Nordic recommendations, especially among men. However, both in men and women, urine potassium excretion was rather high resulting in a Na/K ratio that is more favorable than reported from many other populations. Estimated mean sodium excretion from spot urine by the INTERSALT equation predicted the mean sodium excretion in 24-h urine reasonably well in our Tromsø population.

## Figures and Tables

**Table 1:** Characteristics ^1^ of men and women who collected 24-h urine in the seventh wave of the Tromsø study 2015–2016 (*n* = 493).

	Men (*n* = 241)	Women (*n* = 252)	*p*-Value, Difference ^4^	*p*-Value, Difference ^5^
Age (years) ^2^	56.6 (8.4)	55.2 (8.4)	0.053	0.052
Urine volume (l/24-h) ^2^	1.70 (0.57)	1.79 (0.61)	0.069	0.049
Na (mmol/24-h) ^2^	177.9 (69.7)	129.4 (47.2)	<0.001	<0.001
K (mmol/24-h) ^2^	99.2 (28.4)	76.2 (24.5)	<0.001	<0.001
Na (g/24-h) ^2^	4.09 (1.60)	2.98 (1.09)	<0.001	<0.001
K (g/24-h) ^2^	3.87 (1.11)	2.97 (0.96)	<0.001	<0.001
Na/K-ratio ^2^	1.86 (0.71)	1.79 (0.69)	0.26	0.23
Calculated NaCl (g/24-h) ^2^	10.39 (4.07)	7.55 (2.76)	<0.001	
Calculated NaCl (g/24-h) ^3^	9.73 (2.27–5.0)	7.13 (1.78–18.9)		<0.001
Body Mass Index (kg/m^2^) ^2^	27.6 (3.7)	26.8 (4.7)	0.06	
Tertiary education (%)	51.2	51.4	0.52	

^1^ Sodium (Na), potassium (K), sodium to potassium molar ratio (Na/K ratio). Conversion from mmol to gram is made by multiplying with 0.023 for Na and with 0.039 for K. Conversion from Na to NaCl is made by multiplying with 2.54.^2^ Mean (SD), ^3^ Median (range), ^4^ T-test for means and the chi-squared test for proportions, ^5^ Mann-Whitney U Test (nonparametric test).

**Table 2:** Sodium (Na), potassium (K), and sodium to potassium ratio (Na/K ratio) in 24-h urine by age groups in men and women participating in the seventh wave of the Tromsø Study (*n* = 493), mean and standard deviation.

	N	Na (mmol/24-h) ^1^	K (mmol/24-h) ^2^	Na/K Ratio	Calculated NaCl (g/24-h)
Men					
40–54 years	90	181.2 (71.0)	97.5 (29.5)	1.96 (0.81)	10.6 (4.2)
55–69 years	151	176.0 (69.1)	100.2 (27.7)	1.80 (0.63)	10.3 (4.0)
*p-*value		*0.58*	*0.49*	*0.11*	*0.58*
Women					
40–54 years	112	137.3 (42.1)	74.3 (24.0)	1.97 (0.73)	8.0 (2.5)
55–69 years	140	123.0 (50.3)	77.8 (25.0)	1.65 (0.62)	7.2 (2.9)
*p-*value		*0.02*	*0.26*	*<0.001*	*0.02*

^1^ To convert Na from mmol to gram, multiply by 0.023, ^2^ To convert K from mmol to gram, multiply by 0.039.

**Table 3:** Mean sodium (Na), potassium (K), and sodium to potassium ratio (Na/K ratio) by highest completed education in men and women aged 40–69 years participating in the seventh wave of the Tromsø Study 2015–2016.

	N	Na (mmol/24-h)	K (mmol/24-h)	Na/K Ratio	Calculated NaCl (g/24-h)
Men					
Primary/Secondary	117	177.1	92.0	2.00	10.3
Tertiary, short (<4 years)	67	190.9	106.4	1.87	11.2
Tertiary, long (≥4 years)	56	163.4	105.2	1.56	9.5
*p-*value ^1^		0.09	0.001	0.001	0.09
Women					
Primary/Secondary	122	131.0	74.4	1.86	7.7
Tertiary, short (<4 years)	48	131.6	77.9	1.78	7.7
Tertiary, long (≥4 years)	81	125.7	78.3	1.68	7.3
*p-*value ^1^		0.69	0.48	0.22	0.69

^1^ ANOVA.

**Table 4:** Mean sodium (Na), potassium (K), and sodium to potassium ratio (Na/K ratio) in 24-h urine samples and spot urine samples in 232 men and 243 women aged 40–69 years participating in the seventh wave of in the Tromsø study 2015–2016.

	Na (mmol/l) ^1^	K (mmol/l) ^2^	Na/K Ratio
Men			
24-h urine	111.0 (40.0)	62.5 (20.2)	1.88 (0.70)
Spot urine	110.6 (47.2)	66.5 (26.6)	1.92 (1.12)
*p-*value	*0.84*	*0.06*	*0.49*
Women			
24-h urine	78.6 (34.0)	46.1 (16.5)	1.78 (0.68)
Spot urine	85.5 (47.1)	55.5 (26.1)	1.74 (0.98)
*p-*value	*0.001*	*<0.001*	*0.37*

^1^ To convert Na from mmol to gram, multiply by 0.023, ^2^ To convert K from mmol to gram, multiply by 0.039.

**Table 5:** Mean sodium (Na) excretion measured by 24-h urine and estimated by spot urine in 232 men and 243 women in the seventh wave of the Tromsø study 2015–2016.

	Men, Na (mmol/24-h)		Women, Na (mmol/24-h)	
	Mean	Median	Mean	Median
24-h urine	179.5	168.6	129.6	122.0
INTERSALT, spot urine ^1^	186.5	185.4	128.2	124.7
Toft, spot urine ^2^	195.2	194.1	133.4	133.2
Tanaka, spot urine ^3^	161.8	160.3	154.6	152.4

^1^*p*-value for difference, compared to 24-h urine (mean excretion): *p* = 0.07 in men and *p* = 0.59 in women ^2^ *p*-value for difference, compared to 24-h urine (mean excretion): *p* < 0.001 in men *p* = 0.15 in women ^3^ *p*-value for difference, compared to 24-h urine (mean excretion): *p* < 0.001 in men *p* < 0.001 in women.

## Supplementary Materials

The following are available online at https://www.mdpi.com/2072-6643/11/7/1619/s1, **Table S1.** Equations used in the estimation of 24-h sodium excretion from spot urine sodium, **Figure S1.** Distribution of daily salt intake (NaCl) calculated by one 24-h urine collection in men and women aged 40–69 years participating in the seventh wave of the Tromsø Study 2015–2016 **Figure S2.** Correlation between Na/K ratio in 24-h urine and spot urine samples in 232 men and 243 women in the seventh wave of the Tromsø study 2015–2016. **Figure S3.** Bland-Altman plot—sodium excretion estimated from spot urine by the INTERSALT formula versus sodium excretion measured in 24-h urine in 232 men and 243 women in the seventh wave of the Tromsø study 2015–2016. The vertical line indicates mean sodium excretion.
