# Supplementary material for: Sodium and Potassium Intake Assessed by Spot and 24-h Urine in the Population-Based Tromsø Study 2015–2016
Source: Nutrients. 2019 Jul 16;11(7):1619. doi: 10.3390/nu11071619 (PMC6682958; doi:10.3390/nu11071619)
Supplement: Supplementary file 1 [file nutrients-11-01619-s001.pdf]

Supplementary data

Table 1. Equations used in the estimation of 24-hour sodium excretion from spot urine sodium.

**INTERSALT; Estimated sodium excretion (1):**
Men: 25.46 + (0.46*3 Na_{spot}) – (2.75*Cr_{spot}) – (0.13*K_{spot}) + (4.1*BMI) + (0.26*age) +23.17
Women: 5.07 + (0.34*Na_{spot}) – (2.16*Cr_{spot}) – (0.09*K_{spot}) + (2.39*BMI) + (2.35*age) + (0.03*age^2) + 15.73

**Toft et al; Estimated sodium excretion (2):**
Men, PRCr: -7.54*age + 14.15*weight + 3.48*height (cm) + 423.15
Women, PRCr: -6.13*age + 9.97'weight + 2.45 height (cm) + 342.73
Men, PRNa: 33.56 * (Na_{spot} /(Cr_{spot}*113.1) * $PRCr)^{0.345}$
Women, PRNa: 52.65 * (Na_{spot} /(Cr_{spot}*113.1) * $PRCr)^{0.196}$

**Tanaka et al; Estimated sodium excretion (3):**
PRCr=2.04*age + 14.89*weight (kg) + 16.14 height (cm) -2244.45
PRNa=21.98 * (Na_{spot} /(Cr_{spot}*113.1) * $PRCr)^{0.392}$

Abbreviations:

Cr_{spot}: spot urinary creatinine in mmol/L. *Multiplying Cr_{spot} with 113.1 IN THE Toft and Tanaka formulae in order to transform from mmol/l to mg/l as used in the formulae*

Na_{spot}: spot urinary sodium in mmol/L
K_{spot}: spot urinary portassium in mmol/L
PRCr: Predicted 24-hour urinary creatinine (mg/d)
Weight (kg)
Height (cm)
BMI (kg/m^2)
Age (years)

References to Supplementary table S1

1. Brown IJ, Dyer AR, Chan Q, Cogswell ME, Ueshima H, Stamler J, et al. Estimating 24-Hour Urinary Sodium Excretion From Casual Urinary Sodium Concentrations in Western Populations: The INTERSALT Study. American journal of epidemiology. 2013;177(11):1180-92.
2. Toft U, Cerqueira C, Andreasen AH, Thuesen BH, Laurberg P, Ovesen L, et al. Estimating salt intake in a Caucasian population: can spot urine substitute 24-hour urine samples? European journal of preventive cardiology. 2014;21(10):1300-7.
3. Tanaka T, Okamura T, Miura K, Kadowaki T, Ueshima H, Nakagawa H, et al. A simple method to estimate populational 24-h urinary sodium and potassium excretion using a casual urine specimen. Journal of human hypertension. 2002;16(2):97-103.

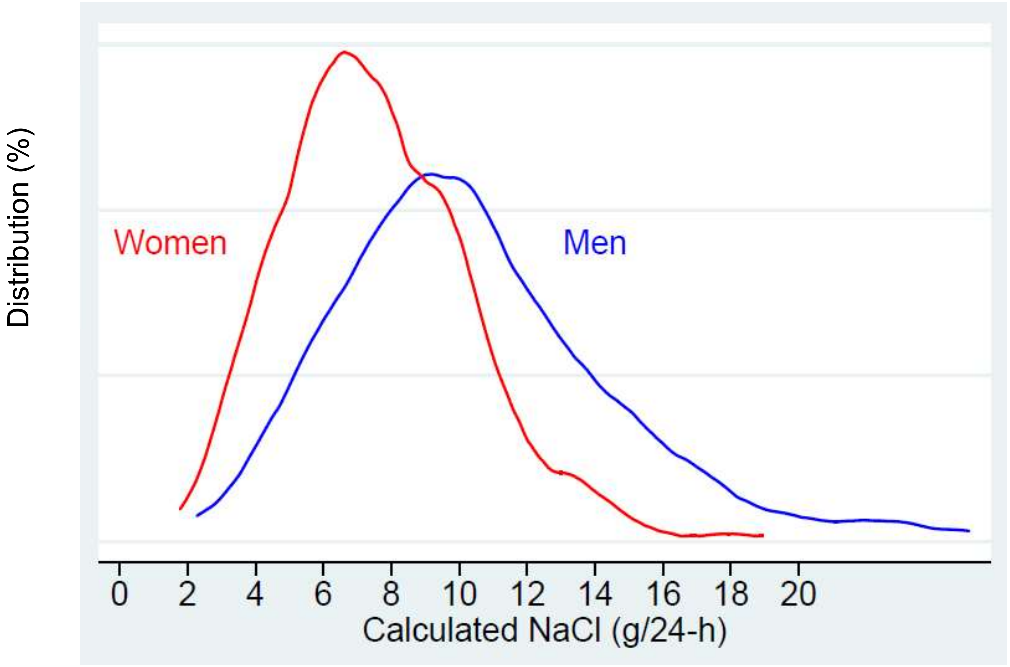

Figure S1. Distribution of daily salt (NaCl) intake calculated by one 24-hour urine collection in men and women aged 40-69 years participating in the seventh wave of the Tromsø Study 2015-16.

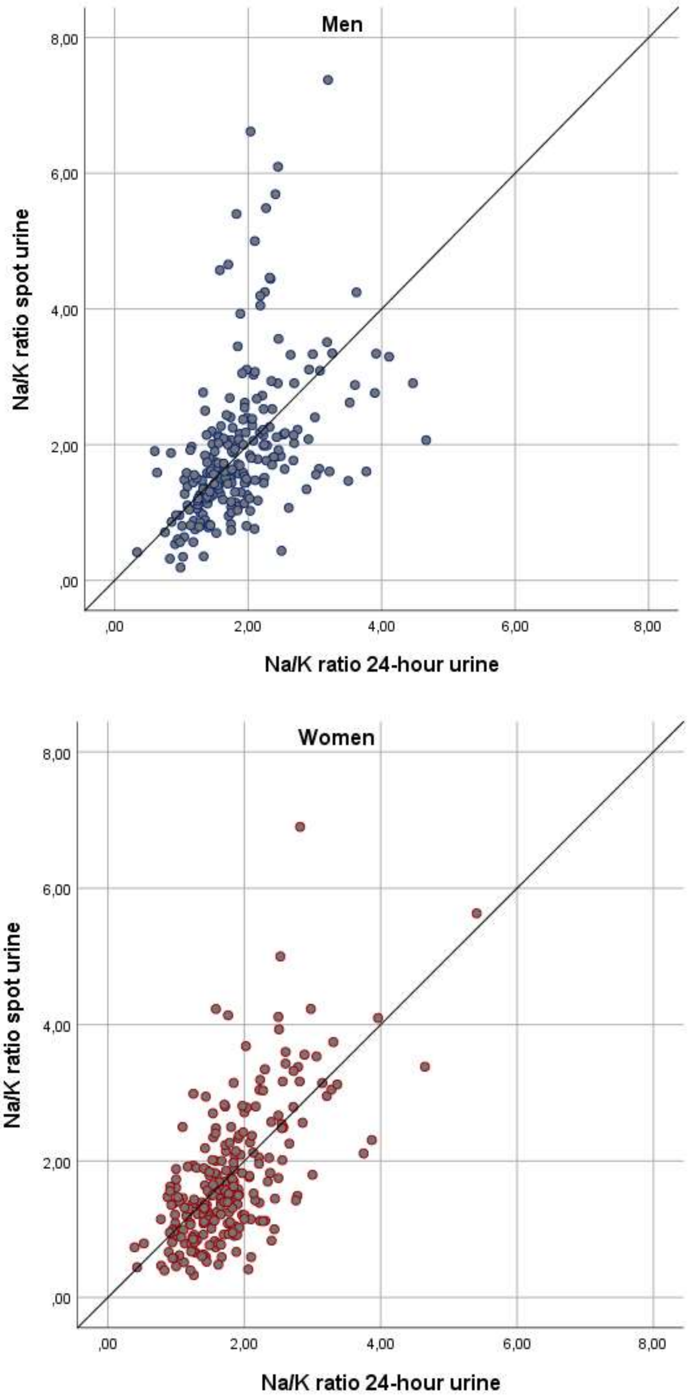

Figure S2. Correlation between Na/K ratio in 24-hour urine and spot urine samples in 232 men and 243 women in the seventh wave of the Tromsø study 2015-16.

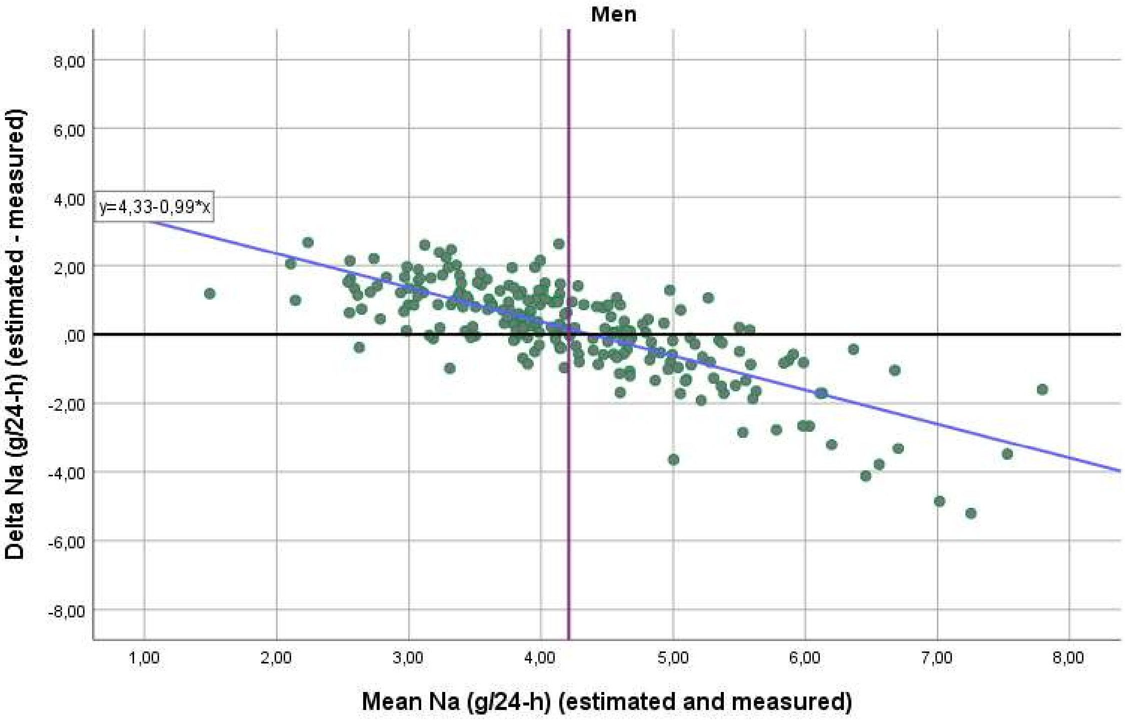

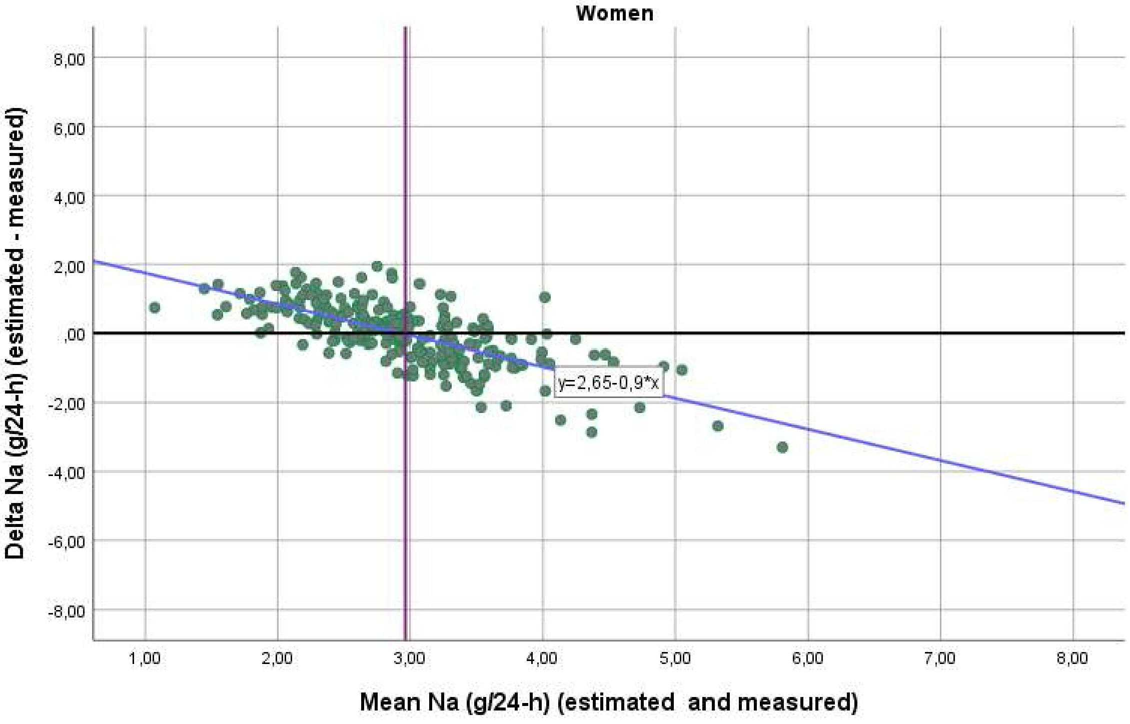

Figure S3. Bland-Altman plot – sodium excretion estimated from spot urine by the INTERSALT formula versus sodium excretion measured in 24-hour urine in 232 men and 243 women in the seventh wave of the Tromsø study 2015-16. The vertical line indicates mean sodium excretion
